# Acute Abdomen Caused by Infected Mesenteric Cyst

**DOI:** 10.5334/jbsr.3555

**Published:** 2024-04-22

**Authors:** Valerie Van Ballaer, Nico Hustings

**Affiliations:** 1 Department of Radiology, UZ Leuven, Leuven, Belgium; 2 Department of Radiology, Sint-Franciscusziekenhuis, Heusden-Zolder, Belgium

**Keywords:** Mesenteric Cyst, Emergency, Infection, CT, MRI

## Abstract

*Teaching point:* Computed tomography is essential for timely diagnosing infected mesenteric cysts as a cause of acute abdomen, ultimately requiring complete excision to confirm diagnosis given the potential of malignant transformation.

## Case

A 21-year-old male presented to the emergency department with sharp peri-umbilical abdominal pain and a loss of appetite for 2 days. Clinical examination revealed mesogastric rebound tenderness and a slightly elevated temperature (37.9°C), with significantly elevated C-reactive protein (220 mg/L).

**Figure 1:**
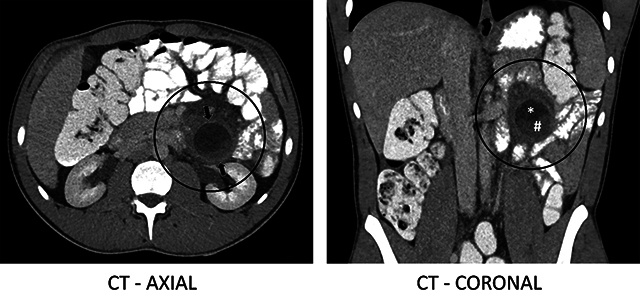
CT images showing the lesion in the left flank.

Single-phase abdominal computed tomography (CT) was performed after the administration of oral and intravenous iodine-based contrast. An intra-mesenteric thin-walled rounded structure of nearly 4 cm was observed in the left flank, anterior to and with a broad base against the anterior pararenal fascia, with infiltration of the surrounding mesenterial fat (Figure 1, circles) and peripheral contrast enhancement (Figure 1, arrows). The content was heterogeneous, with an internal hypodense fluidlike component (Figure 1, *), and a somewhat denser component inferiorly (Figure 1, #) which was not conclusive for eventual enhancement on this single-phase CT.

**Figure 2:**
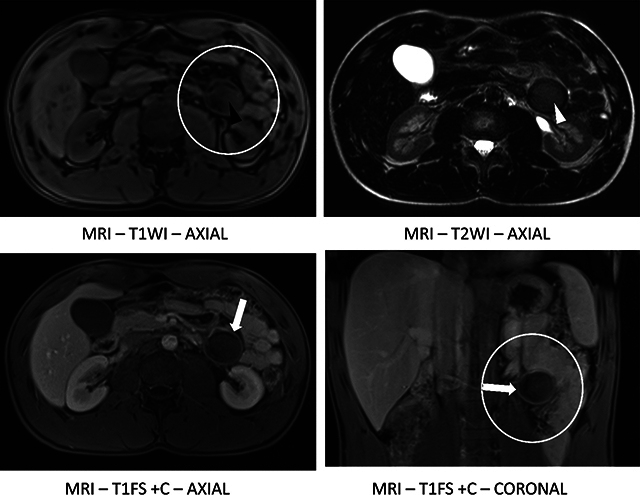
MR images showing the lesion in the left flank.

Magnetic resonance imaging (MRI) with intravenous contrast was performed 6 weeks later. MRI showed persistence of the lesion in the left flank (Figure 2, circles) with peri-lesional fat-stranding. Importantly, MRI confirmed wall enhancement (Figure 2, arrows). The heterogeneous content was slightly hyperintense on T1-WI (Figure 2, black arrowhead) and T2-WI (Figure 2, white arrowhead), with no clear distinction of the internal components, compatible with proteinaceous or hemorrhagic content. The lesion showed a slight diffusion restriction (not shown).

A robot-assisted laparoscopy revealed an intra-mesenteric cyst without signs of local invasion, which was subsequently resected. Histopathologic analysis confirmed purulent and hemosiderin content, indicative of an infected cyst and prior bleeding, with no evidence supporting malignancy. However, the exact subtype of the mesenteric cyst could not be determined due to the absence of an epithelial lining.

## Comment

Mesenteric cysts are rare, thin-walled cysts found in the gastrointestinal tract’s mesentery, with reported incidences of 1/100,000 in adults and 1/20,000 in infants. These cysts may contain serous, hemorrhagic, purulent, or chylous fluid. The cystic lesions have various origins, primarily classified into six groups, with the chylolymphatic type being the most common. Despite being classified as benign entities, they carry a 3% chance of malignant transformation, emphasizing the importance of timely diagnosis [1].

They typically present with vague symptoms such as nausea, vomiting, anorexia, stomach pain, and changes in bowel patterns, with 40% of cases discovered incidentally during routine exams. Rare emergency scenarios include cystic infection, hemorrhage, or rupture.

CT typically reveals a mesenteric thin-walled rounded structure with hypodense fluid-like content and thus has a crucial role in diagnosing complicated mesenteric cysts as a cause of acute abdomen in emergency situations. MRI aids in identifying atypical features such as heterogeneous cystic contents or pericystic changes in the mesenteric fat or neighboring parenchyma.

Ultimately, a complete excision is necessary for histological confirmation and to rule out malignant transformation.
